# Potential prognostic biomarker SERPINA12: implications for hepatocellular carcinoma

**DOI:** 10.1007/s12094-024-03689-w

**Published:** 2024-09-05

**Authors:** Huan Yang, Panpan Kong, Songyu Hou, Xiaogang Dong, Imamumaimaitijiang Abula, Dong Yan

**Affiliations:** 1 The Department of Hepatopancreatobiliary Surgery, The Affiliated Tumor Hospital of Xinjiang Medical University, Urumqi, 830011 Xinjiang China; 2 The Department of Daily Surgery, The Affiliated Tumor Hospital of Xinjiang Medical University, UrumqiXinjiang, 830011 China

**Keywords:** SERPINA12, Bioinformatics, Prognosis, Hepatocellular cancer

## Abstract

**Background:**

Hepatocellular carcinoma (HCC) remains one of the most prevalent malignant tumors, exhibiting a high morbidity and mortality rate. The mechanism of its occurrence and development requires further study. The objective of this study was to investigate the role of SERPINA12 in the diagnosis, prognosis prediction and biological function within HCC.

**Methods:**

The Cancer Genome Atlas (TCGA) data were employed to analyze the relationship between clinical features and SERPINA12 expression in HCC. Kaplan–Meier curves were utilized to analyze the correlation between SERPINA12 expression and prognosis in HCC. The function of SERPINA12 was determined by enrichment analysis, and the relationship between SERPINA12 expression and immune cell infiltration was investigated. The expression of SERPINA12 was examined in 75 patients with HCC using RT-qPCR and immunohistochemistry, and survival analysis was performed.

**Results:**

The expression of SERPINA12 from TCGA database was found to be significantly higher in HCC tissues than in normal tissues and carried a poor prognosis. ROC curve demonstrated the diagnostic potential of SERPINA12 for HCC. The multivariate Cox regression analysis showed that pathologic T stage, tumor status, and SERPINA12 expression were independently associated with patient survival. The SERPINA12 expression was found to correlate with immune cell infiltration. Our RT-qPCR and immunohistochemical analysis revealed high expression of SERPINA12 in tumor tissues. Survival analysis indicated its association with poor prognosis.

**Conclusion:**

SERPINA12 is a promising biomarker for diagnosis and prognosis, and it is associated with immune cell infiltration.

## Introduction

Liver cancer is the fifth most common form of cancer and the fourth leading cause of cancer-related death. The most frequent tumor type is hepatocellular carcinoma (HCC), which accounts for more than 80% of primary liver cancer events worldwide [1]. It is projected that by the year 2040, an estimated 1.3 million people could die from liver cancer, representing a 56.4% increase compared to the figures reported in 2020 [2]. Despite advancements in treatment modalities such as surgery, liver transplantation, and targeted therapies, the overall prognosis of HCC remains poor [3]. Therefore, uncovering the intrinsic mechanisms of HCC, identifying novel potential targets, and pinpointing cancer genes associated with HCC prognosis are essential [4]. This is necessary for the discovery of new molecular biomarkers required for the development of effective diagnostic and therapeutic strategies. Several studies have indicated that the dysregulation of adipokines is associated with cancer development, potentially participating in multiple mechanisms to promote cancer cell progression [5, 6]. SERPINA12, an adipokine discovered in 2005, belongs to the serpin family and is associated with the development of insulin resistance, obesity, and inflammation. It is also known as Vaspin, a serine protease inhibitor derived from visceral adipose tissue [7, 8]. The gene encoding SERPINA12 is located on the long arm of human chromosome 14 (14q32.1) and spans a length of 1245 nucleotides. The adipokine expressed by this gene possesses all typical structural domains of a natural serine protease inhibitor, including nine α-helices, three β-folds, and a flexible reactive center loop (RCL) with a protease recognition sequence at the top [9]. Recent research indicates that the serum and saliva levels of SERPINA12 can serve as biomarkers for the diagnosis of breast cancer in postmenopausal women [10]. Furthermore, elevated serum levels of SERPINA12 have been identified as an independent protective factor for endometrial cancer [11]. Another study showed that the overexpression of SERPINA12 was found to be significantly associated with pancreatic ductal adenocarcinoma compared to benign diseases [12]. Understanding the molecular mechanisms of SERPINA12 dysregulation in HCC is crucial for elucidating its role in tumor development. Through bioinformatics analysis, we investigated the expression of SERPINA12 in HCC and its association with the disease, providing new insights for the diagnosis, pathogenesis, and prognosis of diseases. Experimental validation was also conducted. In summary, SERPINA12 is a promising prognostic biomarker for HCC.

## Material and methods

### Data mining of SERPINA12 in TCGA

We performed data mining from the The Cancer Genome Atlas (TCGA) database (https://portal.gdc.cancer.gov/), which includes both the patients' clinical data and gene expression data. RNA-seq data from 33 tumors was extracted for pan-cancer analysis, focusing on the gene SERPINA12. The dataset related to HCC (LIHC, Liver Hepatocellular Carcinoma, available on the TCGA) consisted of 374 tumor tissues, 50 normal liver tissues, and an additional 50 paired samples. The stats package and car package in R programming language (version 4.2.1) were used to select appropriate statistical methods for data analysis, and the ggplot2 package was used for data visualization.

### Local data collection

From June 2018 to December 2019, a total of 75 individuals meeting the inclusion criteria and free from any exclusion criteria were selected from the clinical and pathological data of the Department of Hepatopancreatobiliary Surgery, the Affiliated Tumor Hospital of Xinjiang Medical University after a rigorous screening process. The inclusion criteria comprised: (1) Patients diagnosed with HCC who underwent their initial surgical treatment. (2) Absence of any pre-surgical treatments. (3) Confirmed diagnosis of hepatocellular carcinoma through pathological examination. (4) Availability of complete clinical and follow-up data. The exclusion criteria encompassed: (1) Receipt of palliative surgical interventions. (2) History of prior surgeries, medical therapies, or radiotherapies. (3) Presence of other significant illnesses or complications. (4) Incompleteness or absence of critical clinical data.

### Analysis of clinicopathological data and prognostic information

Kaplan–Meier survival analyses from the TCGA database were conducted using the survival package and survminer package to estimate survival probabilities over time for the expression of SERPINA12. The database contains RNAseq data from 424 patients with HCC. The patients were divided into two groups (high group 186, low group 187 and missing data 1) based on the median expression level of SERPINA12. Subgroup and stratified analyses were also performed.

Univariate and multivariate Cox proportional hazards models were applied to determine the effect of selected variables on overall survival (OS) and progression free survival (PFS). The rms package and survival package were used for outcome analysis. The nomograms was created using the results of multivariate analysis. It can be employed to predict 1-, 3-, and 5-year survival based on the identified prognostic factors. Calibration was employed to assess and validate the performance of the nomogram in predicting outcomes.

### Functional enrichment analysis and protein–protein interaction (PPI) network construction

Single gene differential expression analysis of SERPINA12 was performed utilizing DESeq2 package and edgeR package. The mRNA gene expression data forHCC was acquired from the TCGA-LIHC dataset. The data was divided into high and low expression groups according to the median expression level of SERPINA12. Then, a comparative analysis was conducted to identify and select differentially expressed molecules within the expression profile of these two groups. The significant differentially expressed genes (DEGs) were identified using established criteria, specifically a logarithmic fold change (log FC) with an absolute value greater than 1 and an adjusted p-value below 0.05. Volcano and heat maps were generated to visually represent the findings of the analysis. Enrichment analysis based on Gene Ontology (GO) and Kyoto Encyclopedia of Genes and Genomes (KEGG) was performed using the clusterProfiler package. GO analysis, which included biological process (BP), cellular component (CC), and molecular function (MF), was used for functional annotation, while KEGG analysis was employed for pathway enrichment analysis. The protein–protein interaction network was performed using STRING (v12.0). We selected the type of full STRING network that includes both functional and physical protein associations. The minimum required interaction score was 0.4, with a maximum of no more than 20 interactors.

### Analysis of immune cell infiltration

In order to assess the relationship between SERPINA12 expression and the infiltrating immune cells, we conducted single-sample gene set enrichment analysis (ssGSEA). The enrichment scores for each immune cell type through ssGSEA were computed using TCGA-LIHC expression data and GSVA package. We utilized the Spearman Wilcoxon rank-sum test analysis via the GSVA package, with statistical significance defined as p < 0.05.

### Immunohistochemistry

The expression of SERPINA12 was analyzed through immunohistochemical analysis in tumor tissues and adjacent tissues from 75 patients with hepatocellular carcinoma (HCC). The collection of all samples was approved by the ethics committee, and informed consent was obtained from the patients. The paraffin-embedded sections were dewaxed with xylene and dehydrated with a gradient alcohol series. The antigen retrieval was performed in sodium citrate (pH 6.0) under high pressure and heat for 10 min. The primary antibody used was rabbit anti-SERPINA12 antibody (No. bs-7536R, Bioss) at a dilution of 1:100, incubated at 37 ℃ for 60 min, and rinsed with phosphate-buffered saline (PBS) three times for 3 min. The secondary antibody (No. PV-6000, ZSGB-Bio) was incubated at 37 ℃ for 20 min and subsequently rinsed three times for 3 min each with PBS buffer. The color reaction was stained with 3, 3ʹ -diaminobenzidine tetrahydrochloride (DAB). The Hematoxylin was used as a counterstain to enhance the visualization of cellular structures in the tissue sections. In the final step, the tissue sections underwent dehydration using an ethanol gradient, clearing with xylene, and mounting with neutral resin.

All cases were evaluated independently by two professional pathologists. The following categories were defined for the evaluation: 0 for no staining, 1 for weak staining, 2 for moderate staining, and 3 for strong staining. The percentage of immunopositive cells was also quantified as follows: 0 for no staining, 1 for 1–25%, 2 for 26–50%, 3 for 51–75%, and 4 for more than 75% of tumor cells stained. The final score was determined by multiplying the score of intensity of staining by the score of the percentage of positive area. Those scores that were equal to or less than 4 were categorised into the negative group, whereas those that exceed 4 were classified into the positive group. The significance of the difference between the two groups was assessed by chi-square test.

### Reverse transcription quantitative polymerase chain reaction (RT-qPCR) analysis

The expression of SERPINA12 in HCC tissues and its relationship with immune cell infiltration were further validated using RT-qPCR. The expression levels of SERPINA12 in tumor tissues and adjacent tissues were analyzed and compared. Additionally, the samples were divided into high and low expression groups based on the median expression level of SERPINA12, and the expression levels of related immune cell marker genes in these groups were analyzed.

This study utilized tissue samples obtained from 75 patients who underwent surgery and were confirmed to have HCC through immunohistochemistry. The frozen liver cancer tissues and adjacent tissues were used for quantitative PCR (qPCR). The frozen samples were immediately frozen in liquid nitrogen after surgery and stored at − 80 °C to preserve RNA integrity for qPCR analysis. Total RNA was extracted from frozen tissue samples. The extraction was performed with TRIzol reagent (Invitrogen, Cat. No. 15596026CN) according to the manufacturer's instructions. Complementary DNA (cDNA) was synthesized from the extracted RNA using the PrimeScript RT Reagent Kit (Takara, Code No. RR037A). The reaction mixture was incubated at 37 °C for 1 h, followed by inactivation of the reverse transcriptase at 85 °C for 10 min. The synthesized cDNA was stored at − 20 °C (Table 1).

**Table 1:** Univariate and multivariate Cox regression analysis conducted on SERPINA12 expression in HCC patients

Characteristics	Subgroup		Overall survival				Progression free interval			
			Hazard ratio (95% CI)				Hazard ratio (95% CI)			
			Univariate	P value	Multivariate	P value	Univariate	P value	Multivariate	P value
Age	≤ 60	> 60	1.205	0.295			0.96	0.783		
			(0.850—1.708)				(0.718—1.284)			
Gender	Male	Female	1.261	0.2			1.018	0.909		
			(0.885—1.796)				(0.747—1.387)			
BMI	≤ 25	> 25	0.798	0.235			0.936	0.673		
			(0.550—1.158)				(0.689—1.272)			
Pathologic T stage	T1-T2	T3-T4	2.598	< 0.001	2.36	< 0.001	2.177	< 0.001	1.414	0.157
			(1.826—3.697)		(1.500—3.714)		(1.590—2.980)		(0.875—2.287)	
Pathologic N stage	N0	N1	2.029	0.324			1.370	0.659		
			(0.497—8.281)				(0.338—5.552)			
Pathologic M stage	M0	M1	4.077	0.017	1.31	0.713	3.476	0.035	1.884	0.307
			(1.281—12.973)		(0.311—5.505)		(1.091—11.076)		(0.559—6.350)	
Histologic grade	G1-G2	G3-G4	1.091	0.636			1.152	0.355		
			(0.761—1.564)				(0.853—1.557)			
Tumor status	Tumor free	With tumor	2.317	< 0.001	2.167	0.001	11.342	< 0.001	14.584	< 0.001
			(1.590—3.376)		(1.359—3.457)		(7.567—17.000)		(8.615—24.689)	
Vascular invasion	No	Yes	1.344	0.163			1.676	0.003	1.529	0.048
			(0.887—2.035)				(1.196—2.348)		(1.004—2.326)	
SERPINA12	Low	High	1.420	0.047	1.716	0.018	1.148	0.349		
			(1.004—2.008)		(1.097—2.683)		(0.859—1.535)			

The primers were designed using the Primer3Plus software, and their specificity was subsequently verified using the NCBI Primer-BLAST tool. Forward and reverse primers were designed targeting specific sequences of the target gene (Table 2). Quantitative PCR (qPCR) was performed using SYBR Green PCR Master Mix (Applied Biosystems, USA). The qPCR conditions were as follows: an initial denaturation at 95 °C for 10 min was followed by 40 cycles of denaturation at 95 °C for 15 s, annealing at 60 °C for 30 s, and extension at 72 °C for 30 s. Detection was performed using an ABI 7500 real-time fluorescent quantitative PCR instrument. Each sample was set up in triplicate. The relative expression level of the target gene was calculated using the 2^(-ΔΔCt) method, with GAPDH (Glyceraldehyde-3-phosphate dehydrogenase) used as the reference gene for normalization.

**Table 2:** RT-qPCR primers of SERPINA12 and immune cell marker genes

Target gene		Sequence (5ʹ—> 3ʹ)
SERPINA12	Forward Primer	GCTGTTCTCCTCACGGTGAA
	Reverse Primer	TCACAGTGCCGGGGTCTATA
CD56 (NCAM1)	Forward Primer	CCTATCCCAGTGCCACGATC
	Reverse Primer	ATCCTCTCCCATCTGCCCTT
CD8α	Forward Primer	CCTTACCAGTGACCGCCTTG
	Reverse Primer	CTAGCTGAGAGGCCAGGAGA
CCR7	Forward Primer	AGCTTCTTCAGTGGCATGCT
	Reverse Primer	CAAGAAAGGGTTGACGCAGC
CD66b (CEACAM8)	Forward Primer	CAGCGTACATCCGGAGACTC
	Reverse Primer	CTAGCTGAGAGGCCAGGAGA
CD123	Forward Primer	CTCAGGGAACACGTATCGGG
	Reverse Primer	CCACCAGCTTGTCGTTTTGG
IL-4	Forward Primer	CAACTGCTTCCCCCTCTGTT
	Reverse Primer	AGCCTTTCCAAGAAGTTTTCCA

### Statistical analysis

This study employed statistical analyses and data visualizations using R software (version 4.3.3). Survival analysis was conducted using standard Kaplan–Meier analysis. The Wilcoxon rank-sum test was applied to non-paired samples, while the Wilcoxon signed-rank test was utilized for the paired samples. The Cox regression was employed for conducting both univariate and multivariate analyses of prognostic factors in the study. The local data were analyzed with SPSS software (version 26.0). All tests were two-sided, and a p-value of less than 0.05 was considered statistically significant.

## Results

### Overexpressed SERPINA12 in HCC tissues

**Fig. 1:**
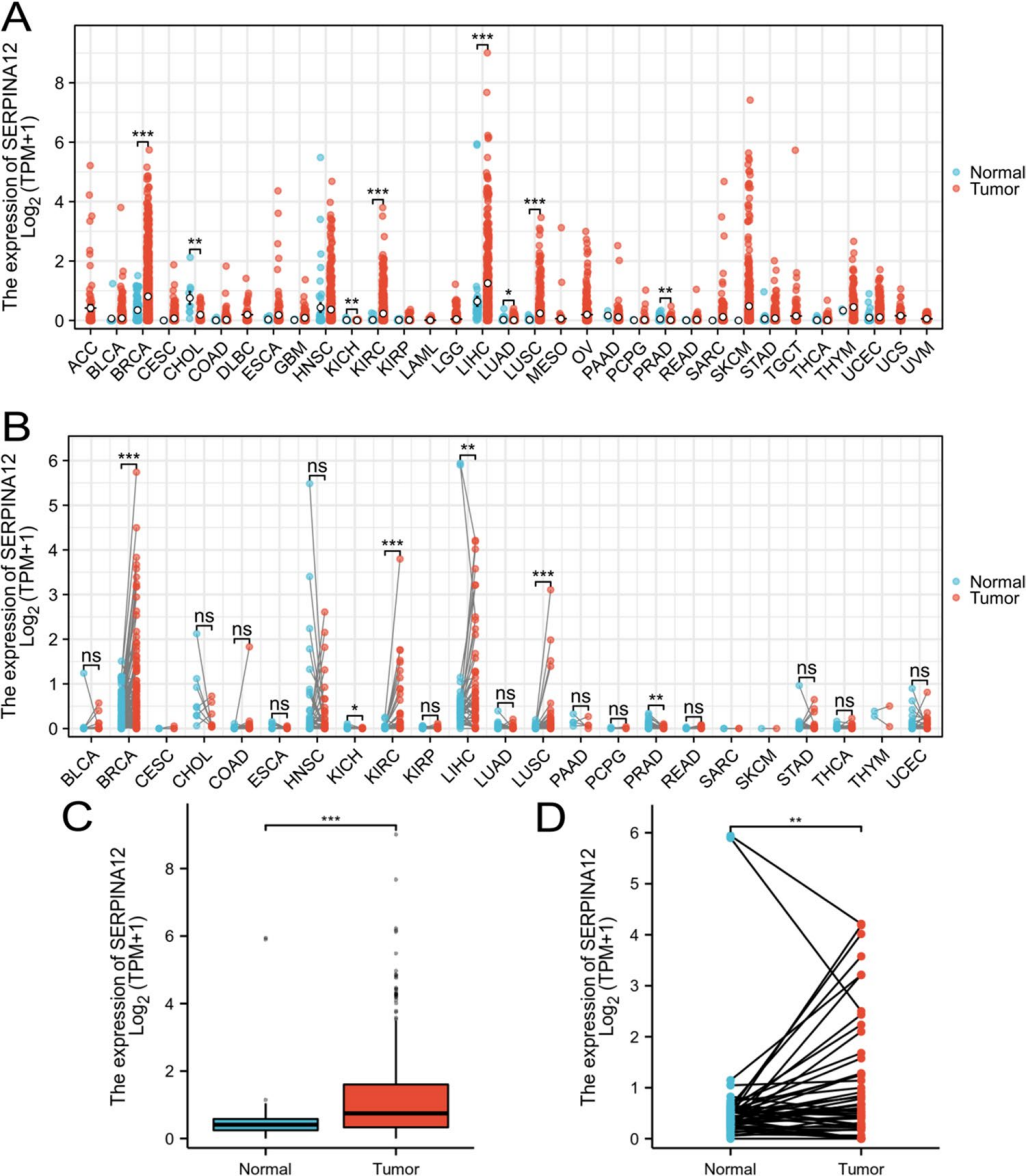
SERPINA12 expression in pan-cancers from TCGA database. **A** Unpaired samples for tumour tissues and non-tumour tissues. **B** Paired samples for tumor tissues and adjacent tissues. **C** Expression of SERPINA12 in the unpaired group of HCC. **D** Expression of SERPINA12 in the paired group of HCC. significances: ns, p ≥ 0.05; *p < 0.05; **p < 0.01; ***p < 0.001

We examined the expression of SERPINA12 using RNA-seq data in 33 cancer types from the TCGA. The results showed that 4 diseases exhibited high expression in tumor tissues, including liver hepatocellular carcinoma (LIHC), breast invasive carcinoma (BRAC), lung squamous cell carcinoma (LUSC) and kidney renal clear cell carcinoma (KIRC). In contrast, 4 diseases exhibited low expression, including cholangiocarcinoma (CHOL), kidney chromophobe (KICH), lung adenocarcinoma (LUAD) and prostate adenocarcinoma (PRAD). These results above were obtained from unpaired samples (Fig. 1A). In the analysis of paired samples, 4 diseases exhibited high expression in tumor tissues, including liver hepatocellular carcinoma (LIHC), breast invasive carcinoma (BRAC), lung squamous cell carcinoma (LUSC) and kidney renal clear cell carcinoma (KIRC). In contrast, 2 diseases exhibited low expression, including kidney chromophobe (KICH) and prostate adenocarcinoma (PRAD). These results above were obtained from paired samples (Fig. 1B). The observed differences are statistically significant in HCC, with a P-value of 0.006 in the paired group and a P-value less than 0.001 in the unpaired group (Fig. 1C and D).

**Fig. 2:**
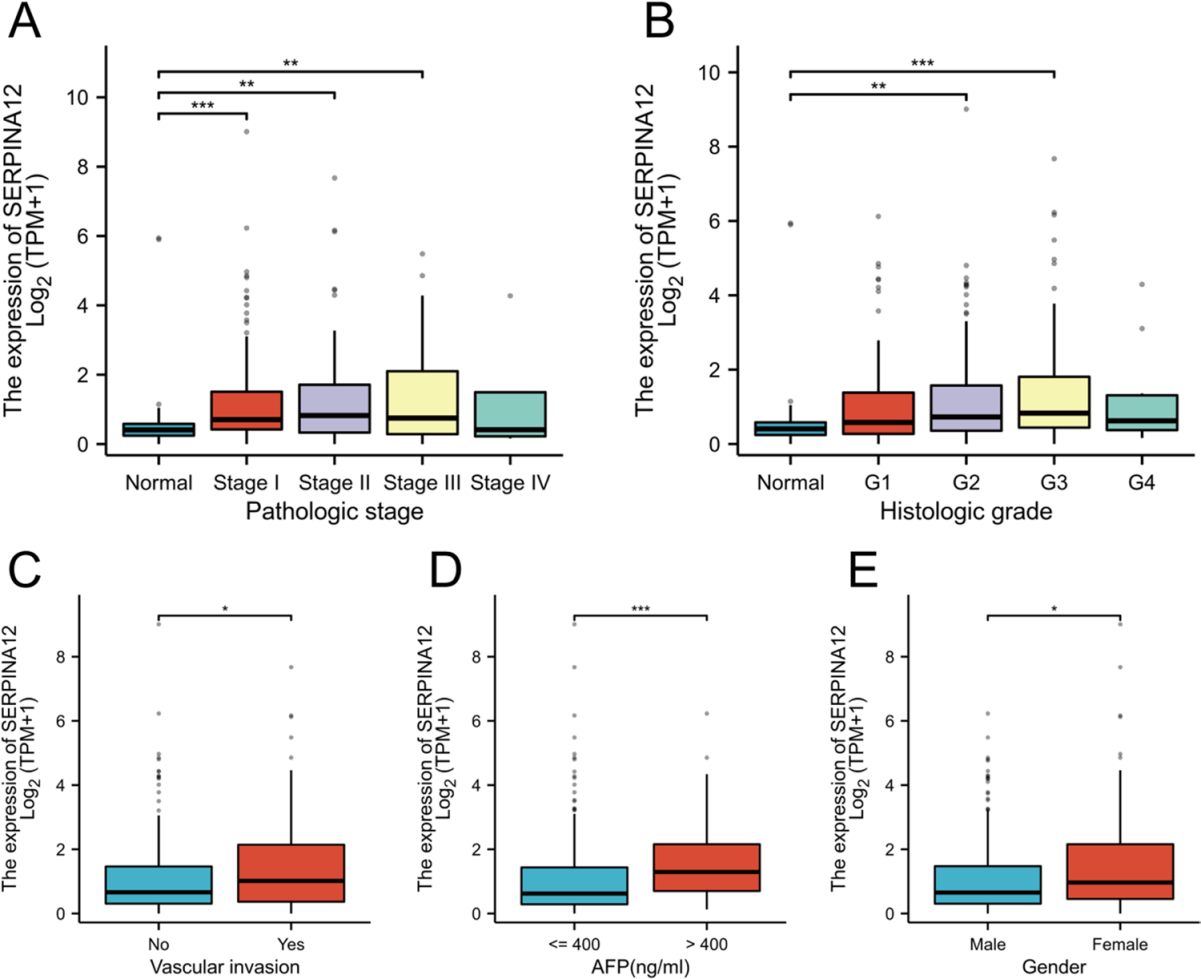
Correlation of SERPINA12 expression with clinicopathologic characteristics. **A** pathologic stage, **B** histologic grade, **C** vascular invasion, **D** AFP level, e gender, ns, p ≥ 0.05; *p < 0.05; **p < 0.01; ***p < 0.001

To evaluate the clinical significance of SERPINA12 in HCC, we analyzed the association between SERPINA12 expression levels and clinicopathological characteristics in the TCGA-LIHC datasets of 374 samples. The results indicated that SERPINA12 expression was positively correlated with individual cancer stages (p < 0.01) and tumor grade (p < 0.01) (Fig. 2A and B). In the comparison of AFP levels with SERPINA12 expression, the AFP (ng/mL) > 400 group exhibited higher expression than the AFP (ng/mL) ≤ 400 group (p < 0.001) (Fig. 2D). In the groups of vascular invasion and gender, their differences are also statistically significant, The expression of SERPINA12 is correlated with vascular invasion (P < 0.05) (Fig. 2C) or female patients (P < 0.05) (Fig. 2E).

### The diagnostic and prognostic significance of SERPINA12 in HCC

**Fig. 3:**
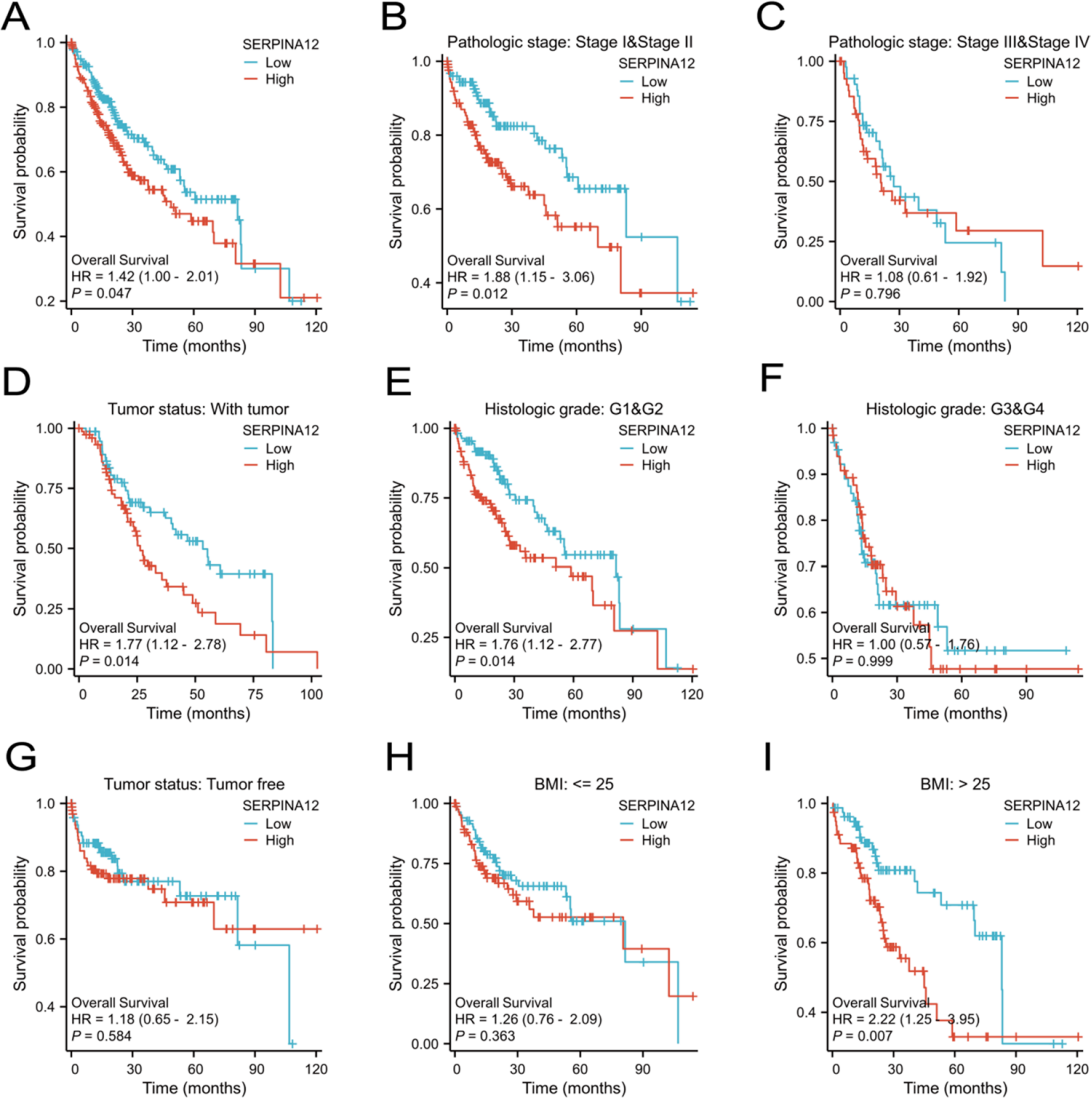
Kaplan–Meier curve and its subgroups in HCC. **A** Overall survival (OS), **B** Pathologic stage I & II, **C** Pathologic stage III & IV, **D** Tumor status: with tumor, **E** Histologic grade: G1&G2, F Histologic grade: G3 & G4, G Tumor status; tumor free, **H** BMI ≤ 25, I BMI > 25

The Kaplan–Meier survival analysis in HCC based on TCGA data revealed a correlation between high SERPINA12 expression and poor overall survival (OS) (p = 0.047) (Fig. 3A). We observed that the differences were more statistically significant in subgroup analyses. These included pathological stage (Stage I and Stage II) (p = 0.012), tumor status (with tumor) (p = 0.014), histologic grade (G1 and G2) (p = 0.014), and body mass index (BMI > 25) (p = 0.007) (Fig. 3B, D, E, I). However, the identified differences were not statistically significant in the subgroups of pathological stage (Stage III and Stage IV), tumor status (tumor free), histologic grade (G3 and G4), and body mass index (BMI ≤ 25) (Fig. 3C, F, G, H). The univariate Cox regression analysis revealed that pathologic T stage, tumor status, and SERPINA12 expression are negative prognostic factors, indicating a worse overall survival (OS) for patients. In the multivariate Cox regression analysis, these variables were found to independently influence overall survival (Table 1). Additionally, progression free interval (PFI) was performed using Cox regression analysis as shown in Table 1.

**Fig. 4:**
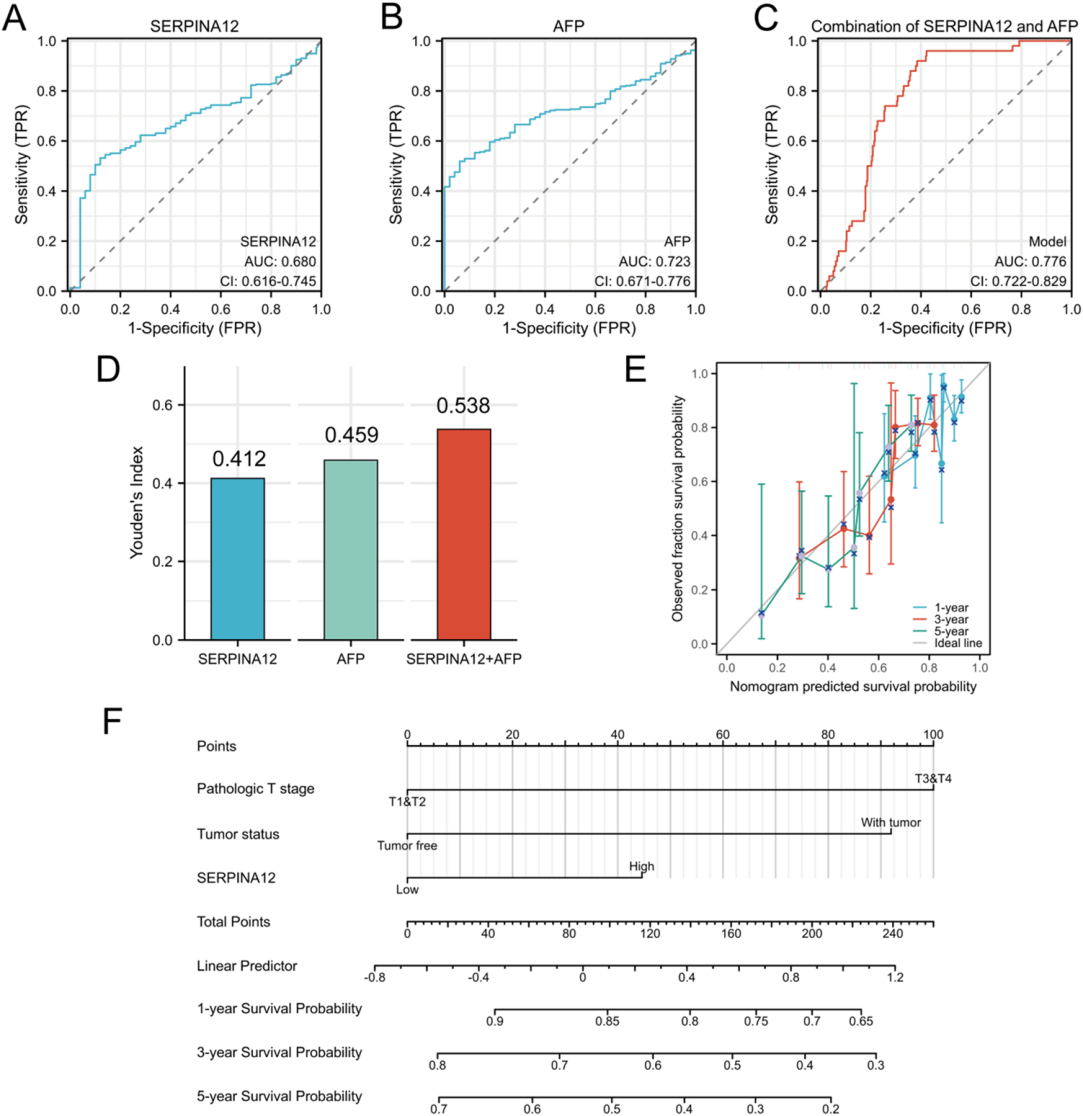
Receiver operating characteristic (ROC) curve and Nomogram for SERPINA12. The ROC curve and the area under the ROC curve was calculated. **A** for SERPINA12, **B** for AFP, **C** for combination of them, **D** Youden’s Index of SERPINA12, AFP, and combination of them, **E** The calibration curves of SERPINA12 for the nomogram, F Nomogram representation of the multivariate model

The potential of SERPINA12 as a biomarker in HCC patients was investigated by evaluating its diagnostic ability using receiver operating characteristics curve (ROC) analysis. The area under the curve (AUC) was calculated, and the result of this calculation is 0.680 (Fig. 4A). Compared with AFP (Fig. 4B), the ability of SERPNIA12 is comparable, and when combined, it can obtain higher values (Fig. 4C). Their Yoden index also shows such patterns (Fig. 4D).

A nomogram was developed based on the findings of a multivariate Cox regression analysis of SERPINA12, which was used to predict the 1-, 3-, and 5-year overall survival rates (Fig. 4F). The calibration plot was employed to calibrate this predictive model (Fig. 4E).

### Enrichment analyses and PPI network

**Fig. 5:**
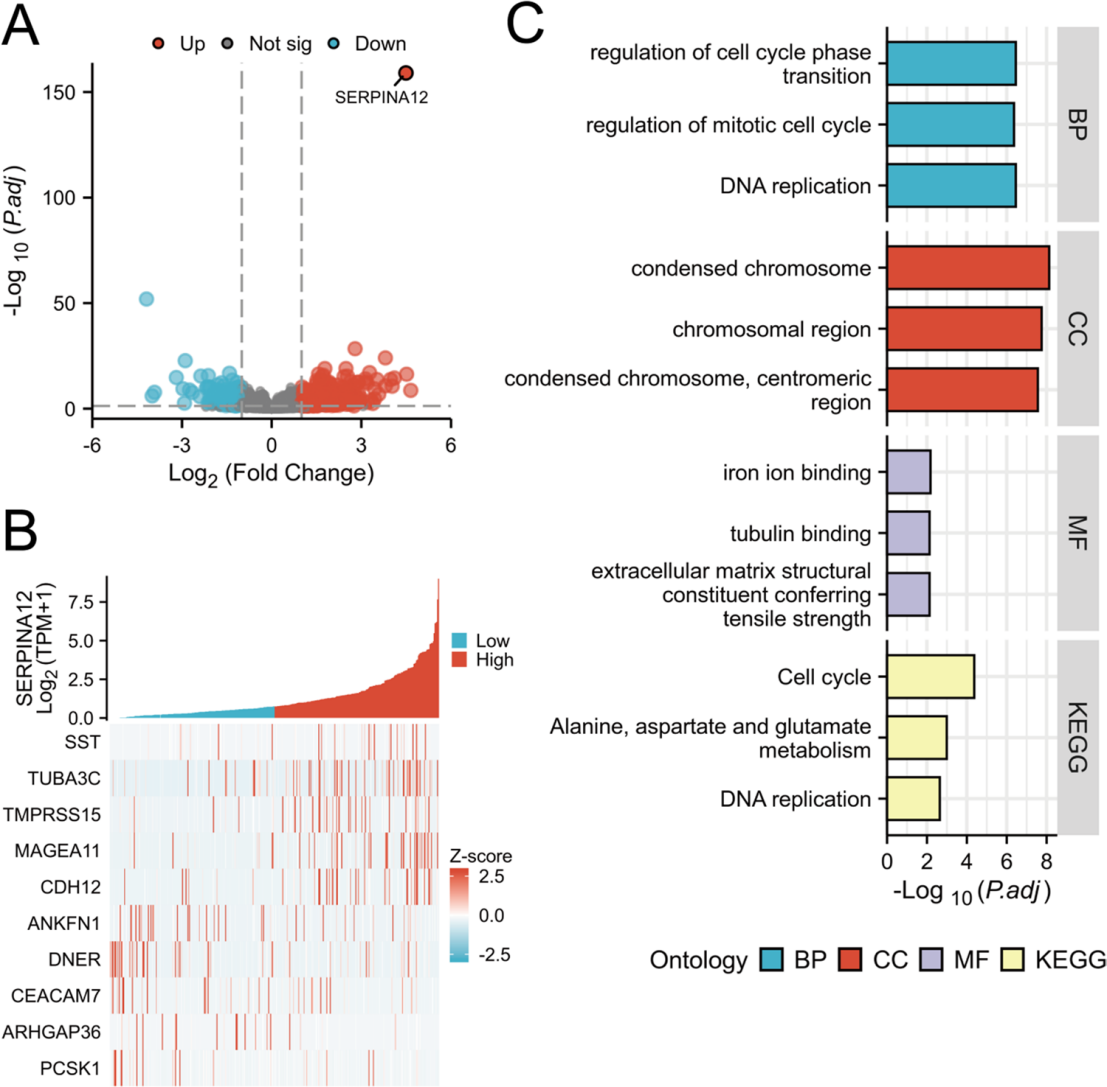
Differential gene expression analysis of SERRPINA12. **A** Volcano map of differentially expressed genes, **B** Heat map of the top 10 most differential genes, **C** Enrichment analysis of SERRPINA12. KEGG, Kyoto Encyclopedia of Genes and Genomes, BP, Biological Process, CC, Cellular Component, MF, Molecular Function

Differential expression analysis identified 18,070 differentially expressed genes from the TCGA-LIHC dataset (|log2(FC)|> 1, P < 0.01; 559 up-regulated and 222 down-regulated genes). The resulting volcano plot illustrates the differentially expressed genes (Fig. 5A). The top 10 (the five up-regulated and five down-regulated) genes were selected for the heat map based on the results of the differential expression analysis (Fig. 5B). GO and KEGG analysis of differential genes was conducted using the clusterProfiler package (Fig. 5C). The results of the GO analysis revealed that in the category of Biological Process (BP), the terms “DNA replication”, “regulation of cell cycle phase transition” and “regulation of mitotic cell cycle” were identified. With regard to the Cellular Component (CC), the terms “condensed chromosome”, “chromosomal region” and “condensed chromosome, centromeric region” were identified. The Molecular Function (MF) category included the terms “iron ion binding”, “extracellular matrix structural constituent conferring tensile strength” and “tubulin binding”. The significant pathways identified in the KEGG analysis include “Cell cycle”, “Alanine, aspartate, and glutamate metabolism”, and “DNA replication”.

**Fig. 6:**
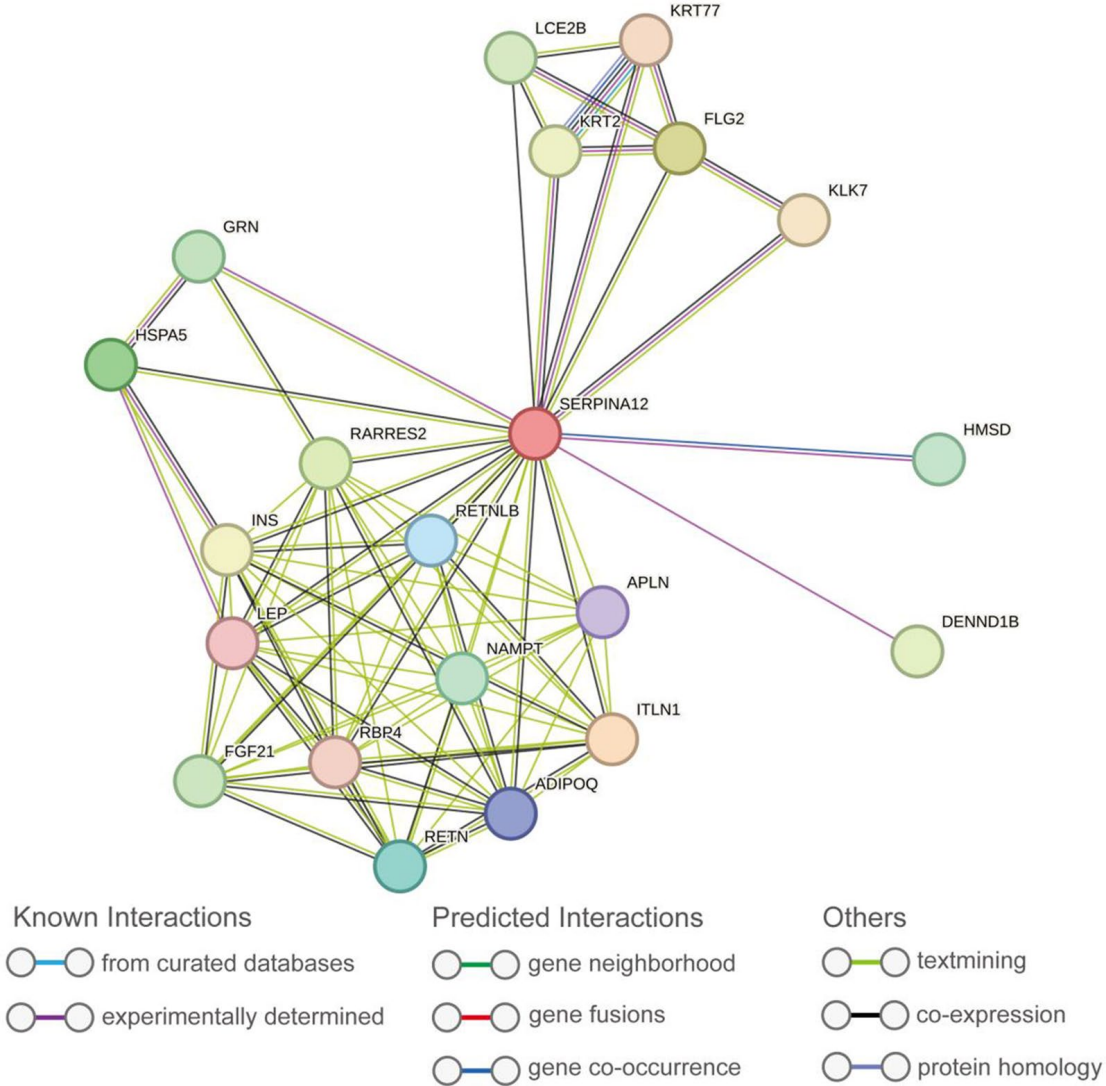
The protein–protein interaction network for SERPINA12 based on STRING

The protein–protein interaction (PPI) network was built and visualized based on the STRING database. A total of 20 proteins associated with SERPINA12 were identified through this analysis (Fig. 6). These proteins are ADIPOQ, APLN,DENND1B, FGF21, FLG2, GRN, HMSD, HSPA5, INS, ITLN1, KLK7, KRT2, KRT77, LCE2B, LEP, NAMPT, RARRES2, RBP4, RETN, RETNLB.

### Relationship between SERPINA12 expression and immune cell infiltration

**Fig. 7:**
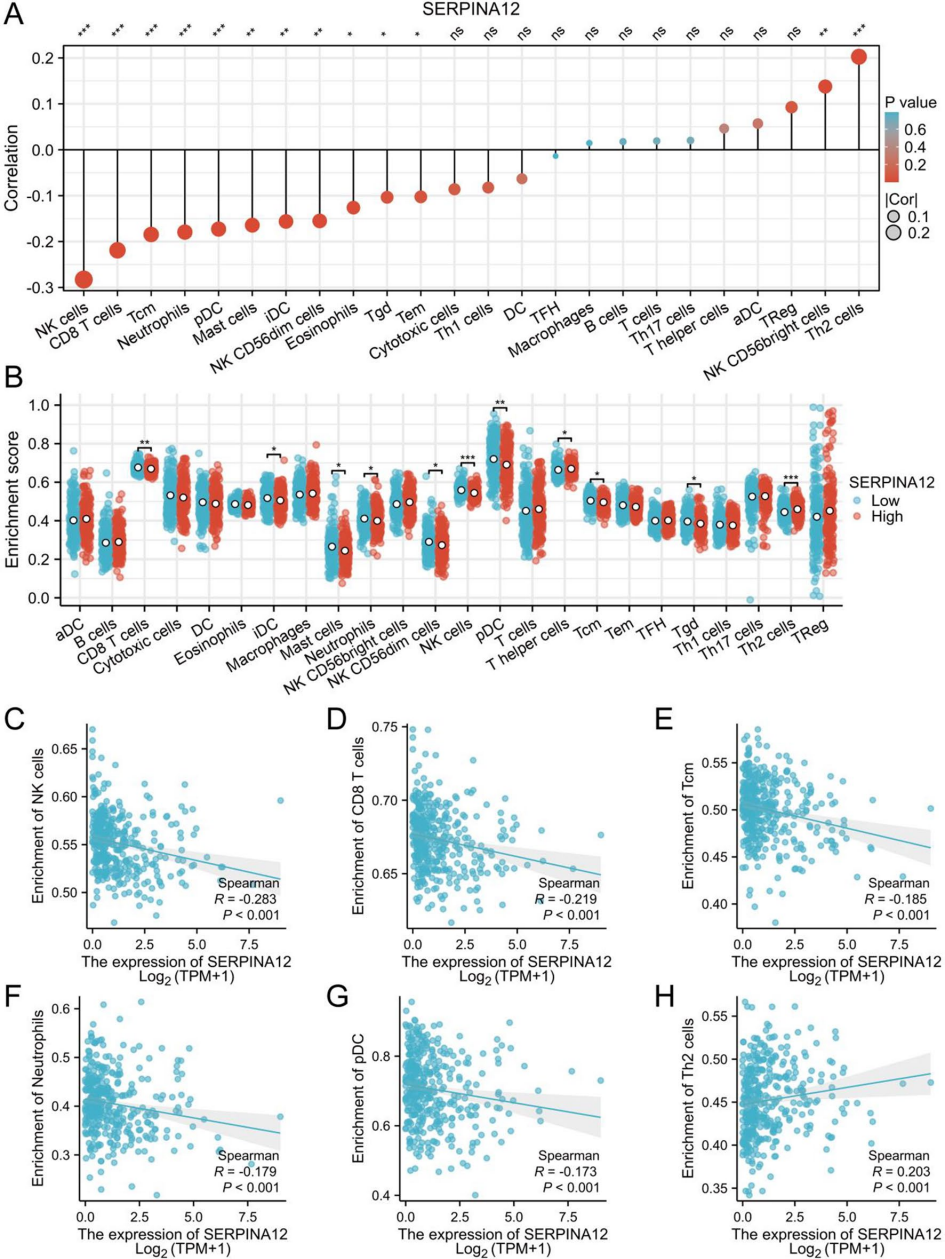
Analysis of immune cell infiltration. **A** Bubble plot of the correlations between SERPINA12 expression and immune cell infiltration level, **B** Subgroups (high and low SERPINA12 expression groups) comparison of immune cell infiltration analysis, Significant infiltrated immune cells: **C** NK cells, **D** CD8 T cells, **E** Tcm, **F** neutrophils, G pDC, H Th2 cells, significances: ns, p ≥ 0.05; *p < 0.05; **p < 0.01; ***p < 0.001

The Spearman correlation analysis was conducted to assess the relationship between SERPINA12 expression and the quantified level of immune cell infiltration measured by ssGSEA. The results demonstrated a positive correlation between SERPINA12 expression and the infiltration of T helper 2 cell (Th2 cells) and NK CD56bright cells and a negative correlation with the infiltration of NK cells, CD8 T cells, T central memory (Tcm), neutrophils, plasmacytoid DC (pDC), mast cells, immature DC (iDC), NK CD56dim cells, Eosinophils, T gamma delta (Tgd) and T effector memory (Tem) (Fig. 7A). We displayed the top six immune cells with the most significant differences through scatter plots (Fig. 7C–H). Furthermore, we attempt to assess the differences in immune cell infiltration and gene expression between the high and low expression groups of SERPINA12 (based on median values) (Fig. 7B). The results demonstrated that the T helper cells, Th2 cells were increased in the high expression group while the CD8 T cells, iDC, mast cells, neutrophils, NK CD56dim cells, NK cells, pDC, Tcm and Tgd were decreased.

### Initial experimental validation

**Fig. 8:**
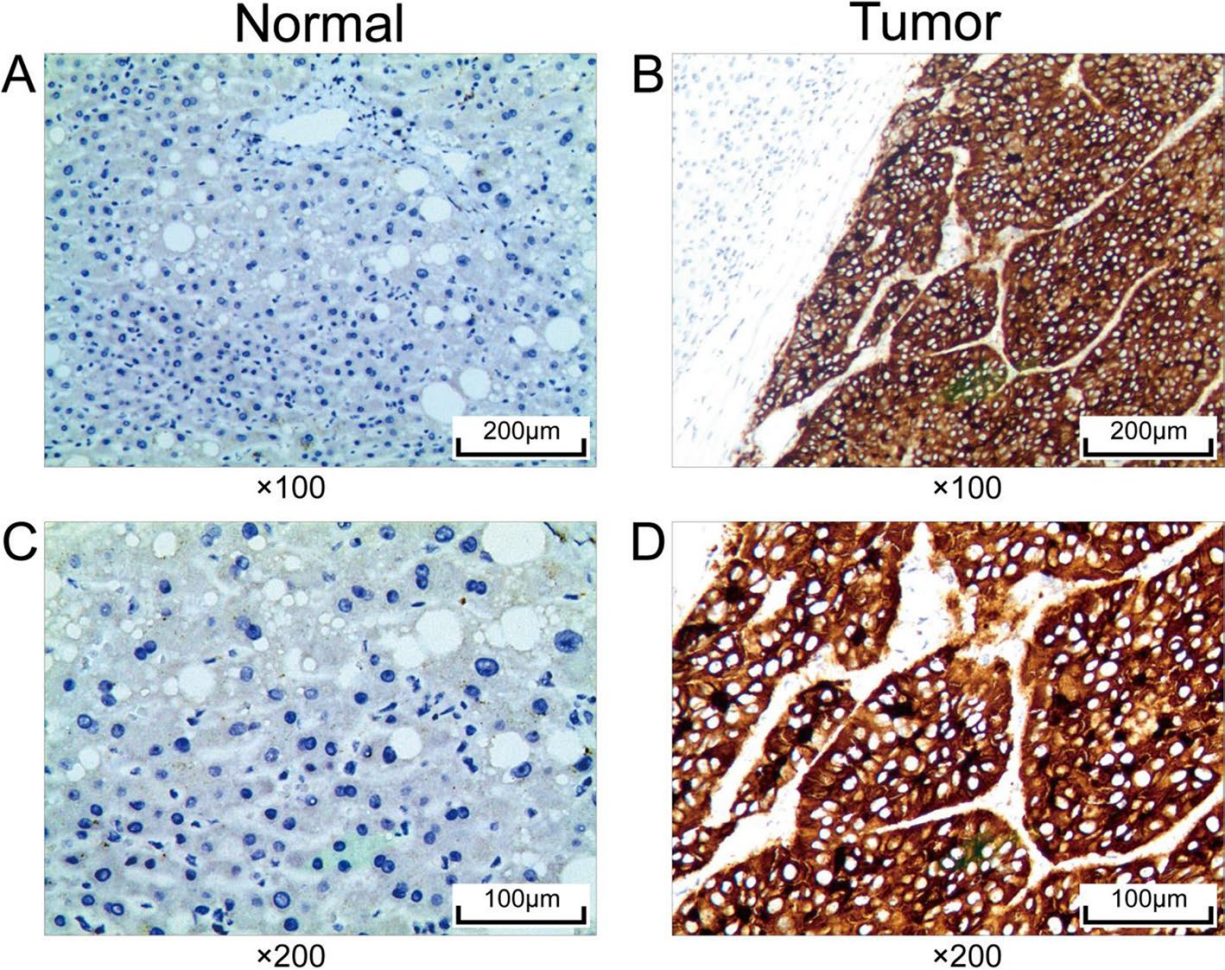
Immunohistochemical staining of SERPINA12 in HCC. **A**, **C** The adjacent tissues were magnified to 100 and 200 times under the microscope. **B**, **D** The tumor tissues were magnified to 100 and 200 times under the microscope

**Fig. 9:**
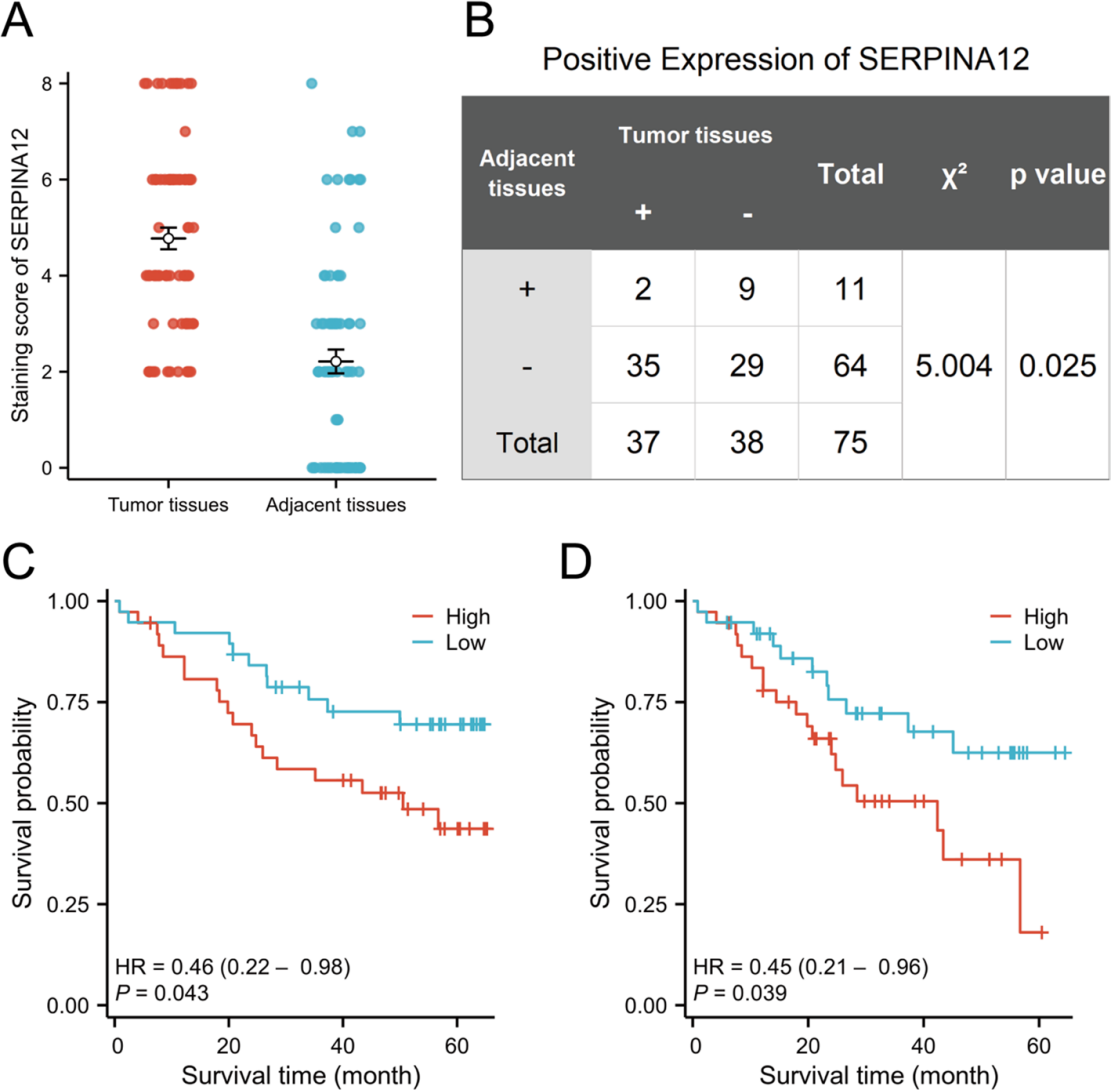
Immunohistochemical analysis SERPINA12 in HCC. **A** Staining score of SERPINA12, **B** Chi-square test for the immunohistochemical data, **C** Kaplan–Meier analysis of 75 patients for overall survival, **D** Kaplan–Meier analysis of 75 patients progression free survival

Staining scores of IHC were determined for 75 paired specimens (Fig. 9A). The immunohistochemical results indicated cytoplasmic expression of SERPINA12, with significantly higher expression levels observed in HCC tissues compared to adjacent tissues (Fig. 8). The positive expression rate of SERPINA12 was 49.3% in cancer tissues and 14.7% in adjacent tissues. These observed differences were statistically significant through a Chi-square test (p = 0.025) (Fig. 9B). Subsequently, Kaplan–Meier survival analyses were performed. The findings validated the association between high SERPINA12 expression and a negative prognosis in 75 patients with HCC (Fig. 9C, D).

**Fig. 10:**
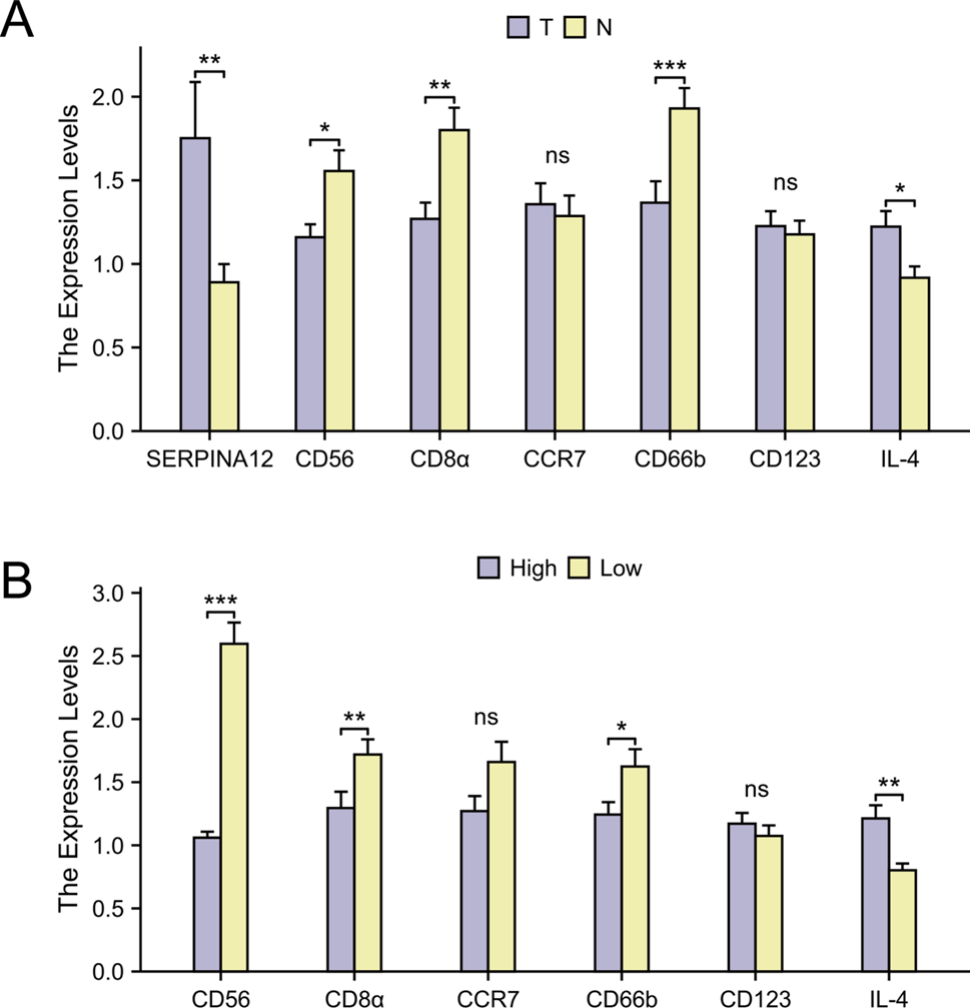
RT-qPCR analysis of SERPINA12 and immune cell marker genes mRNA expression, **A** the groups of tumor tissues and adjacent tissue, **B** the groups of high and low based on the median expression level of SERPINA12

The RT-qPCR results showed that the expression of SERPINA12 was significantly increased in HCC tissues (p = 0.0061) (Fig. 10A). Based on the results of the immune cell infiltration analysis, the six most significant immune cells were selected and their marker genes were analyzed. These immune cell types are NK cells, CD8 T cells, Tcm, neutrophils, pDC, and Th2 cells. Their corresponding immune cell marker genes are CD56 (NCAM1), CD8α, CCR7, CD66b (CEACAM8), CD123 (IL3RA), and IL-4. Compared to adjacent tissues, the expression of CD56, CD8α, and CD66b was significantly decreased in HCC tissues, while the expression of IL-4 was increased. The expressions of CD123 and CCR7 were not statistically significant. When evaluated based on the high and low expression groups of SERPINA12, the expression patterns remained consistent with those observed in the previous analysis (Fig. 10B).

## Discussion

Liver cancer is a global health issue and its burden has increased globally, regionally, and nationally from 1990 to 2019 [13, 14]. HCC is the primary histologic type of liver cancer and accounts for the majority of liver cancer diagnoses and related deaths. [15, 16]. It is often detected in the advanced stages of the disease, when the opportunity for surgical removal has been missed, leading to a poor prognosis. [17]. Surgical resection and liver transplantation are the main methods for treating liver cancer, but intrahepatic metastasis and postoperative recurrence adversely affect the prognosis [18]. Therefore, identification of effective biomarkers and therapeutic strategies is imperative for improving the survival condition of HCC patients. We conducted a comprehensive review of the existing literature on SERPINA12 in relation to cancer. As highlighted in the introduction, the progression of numerous malignant tumors is associated with the abnormal expression of SERPINA12. Through pan-cancer analysis, we have identified further malignancies that are linked to SERPINA12, thus supporting the existing literature findings. It seems that additional research in this field would be advantageous. Our bioinformatics analysis demonstrated that SERPINA12 expression was significantly increased in HCC tissues, suggesting that it may act as an oncogene. In subgroup comparisons, these differences appeared to be more significant in early and middle stages, as well as in other factors such as vascular invasion, high AFP levels, or in women. In survival analysis, high expression of SERPINA12 predicted poor prognosis (OS) with a P value of 0.047. In order to identify more significant differences, we performed subgroup analysis for survival. The results showed that in certain subgroups (pathologic stage I & ll, histologic grade:G1&G2, tumor status: with tumor, body mass index > 25), and the differences were more significant. In summary, high expression of SERPINA12 may play an important role in the development of HCC and predicts a poor prognosis, especially in early and middle stage.

In the single-gene differential expression analysis, we identified the top 10 genes and discovered that some scholars have already conducted related studies on some of them. The somatostatin gene encodes a growth inhibitor and provides the genetic information necessary for the synthesis of growth inhibitors through transcription and translation. It has been proposed that the GH-IGF-SST system may act as a regulator of HCC, directly or indirectly impacting tumor growth and progression by inhibiting cell proliferation, affecting secretion, and inducing apoptosis [19]. Another study showed that the ANKFN1 facilitates the proliferation and metastasis of HCC cells by activating the MEK1/2-ERK1/2 signaling pathway, as evidenced by in vivo and in vitro experiments [20]. Some scholars have also reported that the DNER expression is associated with the proliferation, migration, and invasion of HCC cells. It may potentially be utilized as a prognostic biomarker for HCC [21]. The findings above provide our greater confidence in the ability to predict outcomes and we are hoping for more discoveries.

We noticed that most of the enriched content of GO and KEGG analysis is related to the cell cycle. The cell cycle refers to the entire life cycle of a cell from birth to division, including a series of ordered and complex biological events such as cell growth, DNA replication and division [22]. Some researches pointed out that Cell cycle regulation can enhance tumor cell sensitivity to chemotherapy by inhibiting or promoting tumor cell activity, which may improve cancer treatment outcomes [23]. The cell cycle pathway regulates the sequence of events that a cell goes through to divide and replicate. Disruptions or mutations in this pathway can lead to uncontrolled cell growth, a hallmark of cancer [24, 25]. Another result of enrichment analysis is the iron ion binding of molecular function. Many studies have shown that the changes in iron metabolism play a critical role in cancer progression, as cancer cells require large amounts of iron to proliferate and rely on iron to regulate immune responses [26, 27]. This could be one of the factors contributing to the cancer promoting mechanism of SERPINA12.

The relationship between immune cell infiltration and cancer is crucial. Immune cells play a significant role in recognizing and attacking tumor cells, inhibiting and killing cancer cells. However, sometimes tumors can evade the immune system's attacks through different mechanisms, leading to reduced levels of immune cell infiltration and promoting tumor growth and spread [28, 29]. Our study indicate that the infiltration level of Th2 is significantly correlated with SERPINA12 expression in HCC. In the normal human body, Th1 and Th2 cells maintain a dynamic balance, whereas the imbalance between these two cell types has been implicated as a potential cause of various diseases, including cancer, and their associated complications [30–32]. Some studies have pointed out that the Th2 cells secrete IL-4 and IL-10, which promote tumor growth or metastasis by inducing immunosuppression [33]. Another study also showed that elevated levels of Th2 in breast cancer are associated with invasive characteristics, serving as a potential biomarker for the selection of neoadjuvant therapy in ER-positive breast cancer patients [34]. In the field of hepatocellular carcinoma, there have been reports indicating that elevated levels of Th2 cells may potentially induce this cancer in patients infected with hepatitis C virus (HCV) [35, 36]. In our analysis, certain immune infiltrating cells, including various types of T cells, NK cells, and DC cells, were found to be negatively correlated. However the presence of these cells exerts a suppressive effect on malignant tumors independently [37–39]. High expression of SERPINA12 may potentially diminish their effectiveness. From these observations, we hypothesize that SERPINA12 might play a role in regulating the pathways or functions of these immune cells in the tumor microenvironment. This suggests that further investigation into the role of SERPINA12 in tumor immune evasion and immune suppression could provide valuable insights.

We conducted RT-qPCR and immunohistochemistry experiments and performed survival analysis.These results were consistent with our initial hypothesis, suggesting that SERPINA12 is highly expressed in HCC tissue and is associated with a poor prognosis. This experimental validation provides valuable insights into The study of SERPINA12 in HCC. In our RT-qPCR experiments, we observed a correlation between the expression levels of certain immune cell markers and SERPINA12. These results are consistent with the findings from our previous immune cell infiltration analysis. Although most of the data from our experiments are statistically significant, some results did not achieve significance. Nevertheless, this variability still provides valuable insights and supports our findings, indicating potential trends that warrant further investigation.

It is important to acknowledge the limitations of our experiments, such as small sample size and Limited diversity in experimental design, which may impact the generalizability of the findings. Future research calls for increased sample size and diverse study designs. We can focus on the study of immune cell infiltration to elucidate the underlying mechanisms.

## Conclusion

We conducted bioinformatics analysis and experimental validation to investigate the significance of SERPINA12, proposing it as a promising diagnostic and prognostic biomarker. We observed a correlation between SERPINA12 expression and immune cell infiltration in HCC. Further experiments are needed to study the underlying mechanisms in depth.
